# The Impact of Parental Electronic Health Literacy on Disease Management and Outcomes in Pediatric Type 1 Diabetes Mellitus: Cross-Sectional Clinical Study

**DOI:** 10.2196/54807

**Published:** 2024-03-20

**Authors:** Áron Hölgyesi, Andrea Luczay, Péter Tóth-Heyn, Eszter Muzslay, Eszter Világos, Attila J Szabó, Petra Baji, Levente Kovács, László Gulácsi, Zsombor Zrubka, Márta Péntek

**Affiliations:** 1 Doctoral School Semmelweis University Budapest Hungary; 2 Health Economics Research Center University Research and Innovation Center Óbuda University Budapest Hungary; 3 Pediatric Center Semmelweis University Budapest Hungary; 4 Musculoskeletal Research Unit University of Bristol Bristol United Kingdom; 5 Physiological Controls Research Center University Research and Innovation Center Óbuda University Budapest Hungary

**Keywords:** electronic health literacy, parents, caregivers, diabetes mellitus, child

## Abstract

**Background:**

Despite the growing uptake of smart technologies in pediatric type 1 diabetes mellitus (T1DM) care, little is known about caregiving parents’ skills to deal with electronic health information sources.

**Objective:**

We aimed to assess the electronic health literacy of parents caring for children with T1DM and investigate its associations with disease management and children’s outcomes.

**Methods:**

A cross-sectional survey was performed involving 150 parent-child (8-14 years old with T1DM) dyads in a university pediatric diabetology center. Parents’ electronic health literacy (eHealth Literacy Scale [eHEALS]), general health literacy (Chew questionnaire and Newest Vital Sign [NVS]), and attitudes toward T1DM care (Parental Self-Efficacy Scale for Diabetes Management [PSESDM] and Hypoglycemia Fear Survey [HFS]) were investigated. Children’s treatment, HbA_1c_ level, and quality of life (Pediatric Quality of Life Inventory Diabetes Module [PedsQL Diab] and EQ-5D-Y-3L) were assessed. Multiple linear regression analysis was performed to investigate the determining factors of 6-month average HbA_1c_.

**Results:**

Of the 150 children, 38 (25.3%) used a pen, 55 (36.7%) used a pen plus a sensor, 6 (4.0%) used an insulin pump, and 51 (34.0%) used an insulin pump plus a sensor. Parents’ average eHEALS score (mean 31.2, SD 4.9) differed significantly by educational level (*P*=.04) and the children’s treatment (*P*=.005), being the highest in the pump + sensor subgroup. The eHEALS score showed significant Pearson correlations with the Chew score (r=−0.45; *P*<.001), NVS score (r=0.25; *P*=*.*002), and PSESDM score (r=0.35; *P*<.001) but not with the children’s HbA_1c_ (r=−0.143; *P*=.08), PedsQL Diab (r=−0.0002; *P*>.99), and EQ-5D-Y-3L outcomes (r=−0.13; *P*=.12). Regression analysis revealed significant associations of the child’s HbA_1c_ level with sex (β=0.58; *P*=.008), treatment modality (pen + sensor: β=−0.66; *P*=.03; pump + sensor: β=−0.93; *P*=.007), and parents’ self-efficacy (PSESDM; β=−0.08; *P*=.001).

**Conclusions:**

Significantly higher parental electronic health literacy was found in T1DM children using a glucose sensor. The electronic health literacy level was associated with parents’ diabetes management attitude but not with the child’s glycemic control. Studies further investigating the role of parental electronic health literacy in T1DM children managed at different levels of care and the local context are encouraged.

## Introduction

Type 1 diabetes mellitus (T1DM) is one of the most common chronic diseases in children, with an increasing incidence and prevalence globally [1-3], which poses a significant social and economic burden on pediatric patients, their caregivers, and the society [4-11]. To reduce the effects of these consequences, efficient disease management and treatment strategies are needed.

Pediatric T1DM care has become increasingly technology-driven, with improved therapeutics, such as automated insulin delivery systems and continuous glucose monitoring sensors, being increasingly used in treatment [12-15]. These advanced technologies have a positive effect on disease outcomes as they can facilitate reaching glycemic targets and thus reducing diabetes-related complications [16]. Accordingly, international treatment guidelines recommend the use of the most advanced therapeutics that are readily available, affordable, and considered appropriate for pediatric patients with T1DM to maintain appropriate disease control and improve glycemic outcomes [17,18]. When introducing a new device into the treatment, patient and caregiver training is also recommended for proper device operation and use. Likewise, engagement in disease management and appropriate behavior are key factors to obtain the best results and achieve treatment goals. To meet these expectations, proper education of both patients and caregivers, considering their interest in and barriers to technology uptake, is of utmost importance, along with health literacy improvement, which was found to be associated with diabetes outcomes [18-24].

As a constantly evolving concept, there is a wide range of definitions for health literacy [25]. According to the most common and widely accepted interpretation, health literacy broadly refers to people’s ability to find, evaluate, understand, and use health-related information needed to make appropriate and informed health decisions [26]. As it follows from the definition, parents must have a certain level of health literacy to play an effective role in their child’s disease management and to be able to make informed and responsible health-related decisions [27]. However, as indicated by Sanders et al [28], parents often struggle with understanding their child’s health information owing to its increasing complexity, with approximately one-third of parents in the United States having insufficient health literacy. [29]. Furthermore, a low level of parental health literacy is associated with children’s poor health status [30] and may lead to poor disease outcomes such as insufficient glycemic control [31].

Owing to the recent significant growth in internet use, changing consumer habits, and widespread use of digital products, electronic information sources and even artificial intelligence–based technologies play an increasingly important role in the management of pediatric diabetes [32-35]. In a study by Macken et al [36], 43.5% of families of pediatric T1DM patients with internet access used the internet monthly or more often to find T1DM-related health information. At the same time, new sources pose challenges for parents, who need the appropriate abilities to deal with health information to manage their child’s disease properly [28,37]. In relation, the concept of electronic health literacy has been developed, which, building on general health literacy, can be defined as the ability to seek, find, understand, and appraise health information from electronic sources and apply the knowledge gained to address or solve a health problem [38].

Although the growing importance of health literacy has been accompanied by an increase in the number of tools used to measure it, diabetes-specific instruments are rarely available, which makes it difficult to assess parents’ diabetes-related health literacy in a pediatric setting. In a recent systematic review, out of 19 condition-specific instruments, 8 were diabetes-specific, but all were for use in the adult population, and none of them were designed specifically for T1DM [39]. The authors also identified 11 electronic health literacy measurement tools; however, none of them were diabetes-specific. Furthermore, only 3 instruments that assess parental health literacy were identified, but they were not related to pediatric diabetes. Consistently, in studies examining the association of caregiving parents’ general health literacy with their child’s diabetes outcomes and glucose control, parental health literacy was assessed with general tools such as the Short Test of Functional Health Literacy in Adults (S-TOFHLA) [31,40], Newest Vital Sign (NVS) [41,42], Parental Diabetes Numeracy Test (PDNT) [31], and National Adult Reading Test (NART) [43]. However, despite its increasing significance, the role of parental electronic health literacy in pediatric diabetes has not been investigated extensively, and its associations with the child’s disease characteristics and glycemic outcomes have remained unexplored.

The impact of the therapy on the course of the disease, complications, and overall life expectancy can be seen only over a long period, during which a large amount of data is accumulated. The therapeutic goal is to empower the parents and treat pediatric patients effectively at home rather than in the hospital, which is partly to reduce social costs. Hence, parents have become key players in the management of the disease, and it is therefore necessary to obtain an insight into their role in achieving the desired treatment outcome. Given the increasing use of digital technologies and the fact that many of today’s parents, mainly due to their age, have not received any or sufficient formal training at school on searching and using electronic information, their ability to navigate electronic health information requires particular attention.

In this study, we sought to fill the gap in the knowledge of this area. Our primary aim was to assess the electronic health literacy of the parents of children with T1DM, in light of their general health literacy. Moreover, we intend to investigate the associations of parents’ electronic health literacy with diabetes management (including treatment types, parental self-reported attitudes, and diabetologists’ perceptions) and the child’s disease outcomes (including medical and patient-reported outcomes).

## Methods

### Study Design and Participants

A cross-sectional, noninterventional, single-center survey study was performed in 2021-2022 at a university pediatric diabetology center in Hungary. Parents or caregivers and their children with T1DM attending routine diabetology care were invited to participate. Adult caregivers (≥18 years old) living part-time with the child and children (8-14 years old) diagnosed with T1DM for at least 3 months were included. Respondents were informed that participation was voluntary and that their data would remain anonymous and impersonal and would be used solely for scientific purposes. The survey consisted of 3 modules filled in by parents of the child with T1DM (Module 1), the child with T1DM (Module 2), and the child’s treating diabetologist (Module 3). Module 1 was completed on the Qualtrics online survey platform. Modules 2 and 3 were administered on paper, and the responses were digitized and entered into the Qualtrics system. No personal data were recorded online.

### Ethical Considerations

Written informed consent was obtained from all participants upon entry into the study. Ethical approval was obtained from the Hungarian Medical Research Council (IV/3848-1/2021/EKU; BMEÜ/1620-1/2022/EKU).

### Parents’ Survey (Module 1): Main Characteristics, Health Literacy, and Attitudes Toward the Child’s T1DM

Basic demographic characteristics (sex, age, education, residence, marital status, and employment), household data (income and number of persons living in a household), and childcare circumstances were recorded. Parents’ electronic and general health literacy and their attitudes toward the child’s diabetes were assessed using standard measurement tools (eHealth Literacy Scale [eHEALS], Chew questionnaire, NVS questionnaire, Parental Self-Efficacy Scale for Diabetes Management [PSESDM], and Hypoglycemia Fear Survey [HFS]).

The eHEALS was developed to measure electronic health literacy, which refers to the respondent’s self-assessed confidence; knowledge; and ability to find, understand, and use electronic health information [44]. The self-administered questionnaire contains 8 statements on respondents’ awareness of health resources on the internet (items 1 and 2), internet searching skills (items 3 and 4), appraisal of health resources (items 6 and 7), and use of health information (items 5 and 8). Statements are rated on a 5-point Likert scale (possible answers: 1, strongly disagree; 2, disagree; 3, undecided; 4, agree; 5, strongly agree). Item scores are summed, resulting in a final score of 8-40, with a higher score indicating better eHealth literacy. In this study, the validated Hungarian version of the eHEALS questionnaire was used [45].

The Chew questionnaire is a prescreening tool to identify people with low health literacy. It comprises 3 questions concerning the frequency with which respondents feel confident to fill in forms independently, need help in interpretation, and have problems with understanding hospital documents [46,47]. Response options range from 0 (never) to 4 (always). To calculate the final score (range 0-12), the values of the answers are added together. Higher scores indicate lower health literacy [48].

The NVS questionnaire was developed to identify people with limited health literacy [49,50]. Respondents are presented with a nutrition chart and asked 6 questions. Basic reading comprehension skills and simple mathematical calculations are required to answer. The likelihood that a person has limited health literacy is determined by the number of correct answers as follows: 0-1 correct answers indicate a high likelihood of limited health literacy (50% or more); 2-3 correct answers indicate a possibility of limited health literacy; and 4-6 correct answers indicate adequate health literacy.

The PSESDM questionnaire was developed to assess parents’ confidence in their ability to effectively manage their child’s diabetes [51]. It consists of 8 statements with which the level of agreement can be indicated on a 5-point Likert scale (1 [strongly disagree] to 5 [strongly agree]). The final score is calculated by adding up the scores of responses, resulting in a total score of 8-40. A higher score indicates a parent’s greater confidence in caring for their child’s diabetes.

The HFS measures parents’ fear of their children’s hypoglycemic episodes [52]. The first part assesses the parent’s actions to avoid hypoglycemia and related problems (10 statements), and the second part assesses the parent’s concerns about their child’s hypoglycemic episodes (15 statements). Parents are asked to indicate on a 5-level scale how true the statement is for them (response options: 0 [never] to 4 [almost always]). The final score (range: 0-100) is calculated by adding up the individual scores given for each item. Higher scores indicate a greater fear of hypoglycemia.

### T1DM Children’s Survey (Module 2): Health-Related Quality of Life

To assess health-related quality of life (HRQoL), participating children completed 2 validated measurement tools (Pediatric Quality of Life Inventory [PedsQL] and its Diabetes Module [PedsQL Diab] and EQ-5D-Y-3L) for evaluating their general and diabetes-specific quality of life.

The general module of the 23-item PedsQL assesses the following domains: physical functioning (“my health and activities” involving 8 questions), emotional functioning (“my feelings” involving 5 questions), social functioning (“my relationships with others” involving 5 questions), and school functioning (“school” involving 5 questions) [53,54]. Questions are asked for the past month, and responses are given on a 5-point Likert scale (possible answers: 0, never; 1, rarely; 2, sometimes; 3, often; 4, almost always). To calculate the final score, the answers to each question are transformed into a scale from 0 to 100 by inverse scoring (ie, the score for each answer is 0=100, 1=75, 2=50, 3=25, and 4=0), and then, the simple arithmetic average of the scores obtained for each answer is taken. A higher score indicates a better HRQoL.

Version 3.0 of the diabetes module of the PedsQL consists of 28 items and covers the following domains: symptoms of diabetes (“about my diabetes” involving 11 questions), difficulties with treatment (“treatment I” involving 4 questions), acceptance of treatment (“treatment II” involving 7 questions), worry about the disease (“concerns” involving 3 questions), and difficulties with communication (“communication” involving 3 questions) [55]. Response options and the evaluation of the questionnaire are the same as described for the general module. For both the general and diabetes modules, the validated Hungarian version of the questionnaire was used in this study [56,57].

EQ-5D-Y-3L is specifically designed to assess children’s and adolescents’ general HRQoL [58,59]. Its descriptive part contains 5 questions covering the following domains: mobility; taking care of myself; doing usual activities; feeling pain or discomfort; and feeling worried, sad, or unhappy. Each domain is rated on a 3-point Likert scale (1, no problems; 2, some problems; 3, a lot of problems). In this study, EQ-5D-Y-3L index values were calculated using the Hungarian value set [60]. EQ-5D-Y-3L includes a visual analog scale (EQ VAS) on which respondents can indicate their current health status on a vertical scale ranging from 0 (worst “health you can imagine”) to 100 (best “health you can imagine”).

### Diabetologists’ Survey (Module 3): Children’s T1DM Disease Characteristics, Perceptions of Parents, and Disease Management

The following medical information was collected from treating diabetologists: child’s weight, height, duration of disease, duration of care at the center, route of insulin administration and blood glucose measurement (treatment modalities: pen without sensor, pen plus sensor, pump without sensor, and pump plus sensor), HbA_1c_ level, T1DM-related serious acute events (hypoglycemia, hyperglycemia, or other events requiring medical intervention or acute hospitalization) or device malfunction in the last 3 months, chronic complications, and comorbidities. Actual and 6-month average HbA_1c_ levels were recorded as percentage. Treating diabetologists were also asked about the management of T1DM (parents’ cooperation, diabetes knowledge, and knowledge of device use; T1DM being difficult to treat) with responses given on a visual analog scale (VAS), with 0 indicating the worst option and 10 indicating the best option.

### Statistics

Variables were analyzed with descriptive statistical methods (mean, SD, minimum, maximum, and number of items). The average eHEALS score of the study sample was compared to the previously published Hungarian population norm with the Welch test. The effect size was measured with Cohen *d* (small effect=0.2; medium effect=0.5; large effect=0.8) [61]. Two-way ANOVA was carried out to test differences by sex, age, and education.

Subgroup comparisons by sociodemographics, treatment modalities, and T1DM complications were performed with the Welch and ANOVA tests.

Correlations between eHEALS and other measures were assessed by calculating the Pearson correlation coefficient (>0.5=strong; 0.5-0.3=moderate; <0.3=weak) [62].

Multiple linear regression analysis was performed to investigate the factors determining glucose control (6-month average HbA_1c_). A total of 9 regression models were developed to examine the associations of variables and changes in model performance. The following explanatory variables were included: T1DM children’s characteristics (Model 1); parents’ demographic characteristics (Model 2); treatment modalities (Model 3); parental electronic and general health literacy (Model 4: eHEALS; Model 5: Chew; Model 6: NVS); and parents’ self-reported attitudes toward their child’s illness (Model 7: PSESDM; Model 8: HFS).

The model construction was systematic so that the variables included in Models 1 to 3 were included in all subsequent models, while for Models 4 to 8, the variables mentioned above were included one by one in a mutually exclusive manner. The final Model 9 included all variables together.

Statistical analysis was performed using Stata 17 software (StataCorp LCC).

## Results

### Parents’ Main Characteristics, Health Literacy, and Attitudes Toward Their Child’s T1DM

Altogether 150 parent-child dyads were involved in the study. Parents’ mean age was 42.5 (SD 5.8; range: 19-62) years, and 80.0% (120/150) were women. The sociodemographic characteristics are summarized in Table 1. Only 2 (1.3%) caregivers were not parents, and the majority (144/150, 96.0%) lived together with the child full-time in the same household. Moreover, 10 (6.7%) parents had diabetes mellitus themselves.

**Figure 1:**
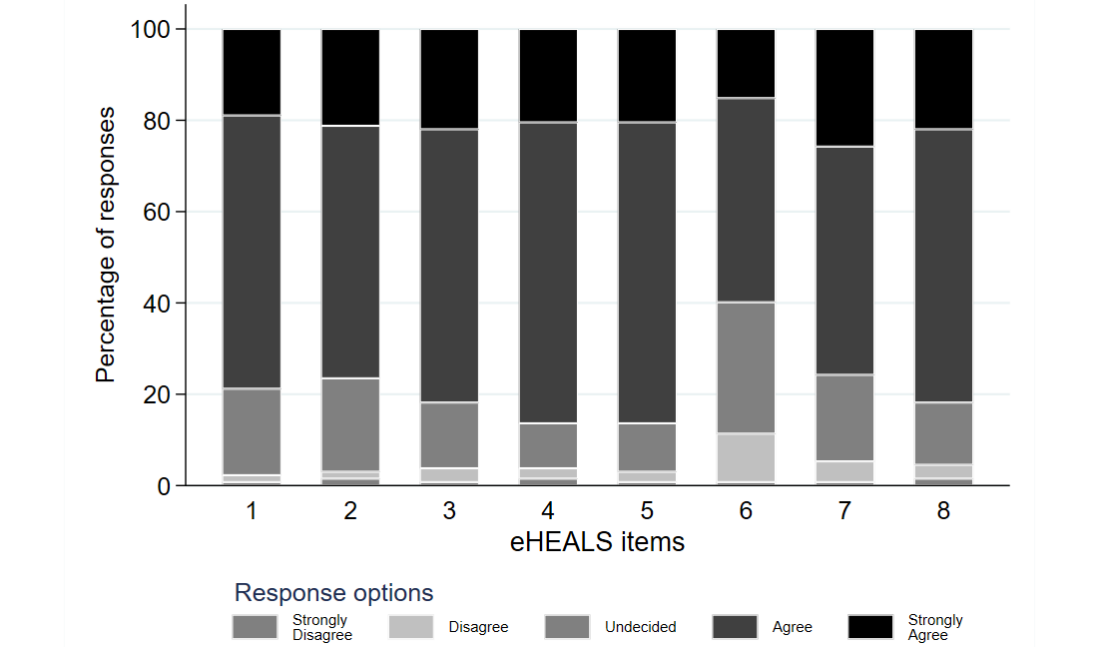
Distribution of responses by eHealth Literacy Scale (eHEALS) items. 1: “I know what health resources are available on the internet;” 2: “I know where to find helpful health resources on the internet;” 3: “I know how to find helpful health resources on the internet;” 4: “I know how to use the internet to answer my questions about health;” 5: “I know how to use the health information I find on the internet to help me;” 6: “I have the skills I need to evaluate the health resources I find on the internet;” 7: “I can tell high-quality health resources from low-quality health resources on the internet;” 8: “I feel confident in using information from the internet to make health decisions” [44].

The distribution of responses by eHEALS items is presented in Figure 1 [44]. All 150 parents responded to all items. The proportion of “strongly agree” responses varied between 15.3% (23/150) and 26.0% (39/150) (mean 20.7%, SD 3.0%) across the 8 eHEALS items, indicating remarkable uncertainty of parents dealing with electronic health information resources, especially on having “the skills I need to evaluate the health resources I find on the internet” (item 6).

**Table 1:** Parents’ demographics, electronic and general health literacy, and attitudes as a caregiver for a child with type 1 diabetes mellitus.

Variable			Value (N=150), n (%)^a^		eHEALS^b^ (score range: 8-40)					Chew (score range: 0-12)^c^					NVS^d^ (score range: 0-6)					PSESDM^e^ (score range: 8-40)					HFS^f^ (score range: 0-100)^g^		
					Score, mean (SD)		*P* value^h^		Score, mean (SD)			*P* value^h^		Score, mean (SD)			*P* value^h^		Score, mean (SD)			*P* value^h^		Score, mean (SD)			*P* value^h^
**Sex**							.40					.41					.14					.36					.07
	Male	30 (20.0)		31.8 (4.3)				2.3 (1.6)					4.8 (1.5)					33.6 (4.7)					29.7 (13.1)				
	Female	120 (80.0)		31.0 (5.0)				2.6 (2.1)					4.3 (1.8)					32.7 (5.6)					34.7 (12.0)				
**Age group (years)**							.50					.58					.09					*.*13					*.*55
	18-24	1 (0.7)		29.0 (0.0)				2.0 (0.0)					2 (0.0)					40.0 (0.0)					25.0 (0.0)				
	25-34	13 (8.7)		29.5 (6.1)				3.2 (2.9)					3.4 (1.9)					31.2 (7.1)					35.7 (12.4)				
	35-44	72 (48.0)		31.8 (4.5)				2.4 (1.8)					4.5 (1.8)					32.6 (5.2)					35.0 (13.0)				
	45-54	62 (41.3)		30.9 (5.0)				2.5 (2.0)					4.6 (1.7)					33.6 (5.2)					31.9 (11.4)				
	55-64	2 (1.3)		29.0 (1.4)				4.0 (0.0)					3.5 (2.1)					26.5 (0.7)					30.5 (20.5)				
**Education (missing=1)**							*.*04					*<.*001					*<.*001					*.*006					*.*24
	Primary	15 (10.0)		29.5 (5.6)				3.1 (2.9)					2.7 (1.7)					29.5 (6.9)					31.7 (11.0)				
	Secondary	70 (46.7)		30.5 (4.7)				3.0 (1.8)					4.0 (1.8)					32.4 (5.2)					32.2 (11.5)				
	Tertiary	64 (42.7)		32.3 (4.7)				1.7 (1.5)					5.4 (1.1)					34.2 (4.9)					35.6 (13.4)				
**Residence**							*.*71					*.*57					*.*002					*.*50					*.*09
	Capital	39 (26.0)		31.1 (4.8)				2.3 (1.7)					4.9 (1.6)					33.4 (5.4)					36.3 (12.4)				
	Town	79 (52.7)		31.0 (5.1)				2.5 (1.9)					4.6 (1.6)					33.0 (5.2)					31.6 (11.3)				
	Village	32 (21.3)		31.8 (4.2)				2.8 (2.4)					3.5 (2.0)					31.9 (6.0)					35.6 (14.0)				
**Living in a relationship**							*.*58					*.*55					*.*87					*.*34					*.*56
	Yes	128 (85.3)		31.3 (4.9)				2.5 (2.0)					4.4 (1.8)					33.0 (5.4)					33.9 (12.7)				
	No	22 (14.7)		30.7 (4.5)				2.8 (2.0)					4.5 (1.8)					31.9 (5.3)					32.4 (10.4)				
**Paid work**							*.*14					*.*45					*.*11					*.*20					*.*19
	Yes	145 (96.7)		31.4 (4.8)				2.5 (1.9)					4.5 (1.7)					33.0 (5.3)					33.4 (12.3)				
	No	5 (3.3)		26.4 (6.0)				3.6 (3.0)					2.4 (2.3)					28.0 (7.4)					41.8 (11.8)				
**Monthly net income per capita (missing=34)**							*.*05					*.*03					*<.*001					*<.*001					*.*58
	1st quintile	24 (16.0)		29.5 (5.7)				3.3 (2.5)					3.1 (1.7)					29.5 (5.2)					34.7 (10.6)				
	2nd quintile	17 (11.3)		29.7 (4.4)				3.3 (1.3)					3.9 (1.7)					31.5 (4.7)					30.6 (9.5)				
	3rd quintile	17 (11.3)		31.1 (3.6)				2.5 (1.6)					4.9 (1.4)					32.0 (5.0)					32.6 (13.9)				
	4th quintile	3 (2.0)		30.7 (2.3)				3.7 (3.5)					3.3 (2.1)					30.0 (6.0)					26.0 (8.0)				
	5th quintile	55 (36.7)		32.5 (4.5)				2.0 (2.2)					5.2 (1.3)					34.3 (4.1)					35.0 (14.2)				
**Living in the same household with the T1DM^i^ child**							*.*40					*.*19					*.*23					*.*75					*.*07
	Full-time	144 (96.0)		31.1 (4.9)				2.6 (2.0)					4.4 (1.8)					32.8 (5.4)					33.8 (12.6)				
	Part-time	6 (4.0)		32.8 (4.5)				1.8 (1.2)					5.2 (1.3)					33.5 (4.7)					29.8 (4.1)				
**Having diabetes**							*.*84					*.*87					*.*95					*.*42					*.*46
	Yes	10 (6.7)		30.8 (6.3)				2.4 (2.6)					4.4 (2.2)					30.9 (7.8)					36.8 (13.5)				
	No	140 (93.3)		31.2 (4.8)				2.5 (1.9)					4.4 (1.7)					33.0 (5.2)					33.4 (12.3)				
Total sample					31.2 (4.9)				2.5 (2.0)					4.4 (1.8)					32.9 (5.4)					33.7 (12.3)			

^a^Percentages may not add up to 100% due to rounding.

^b^eHEALS: eHealth Literacy Scale.

^c^Higher scores indicate lower literacy levels.

^d^NVS: Newest Vital Sign.

^e^PSESDM: Parental Self-Efficacy Scale for Diabetes Management.

^f^HFS: Hypoglycemia Fear Survey.

^g^Higher scores indicate greater fear of hypoglycemia.

^h^Differences between groups were compared using Welch and ANOVA tests.

^i^T1DM: type 1 diabetes mellitus.

In the total sample, the average parental eHEALS score was 31.2 (SD 4.9), which was significantly higher (*P=.*002) than that in the Hungarian general population [45]. The effect size was small, with a Cohen *d* of 0.41 (95% CI 0.16-0.67). Differences were observed between the study sample and the general population by sex, age group, and education, but the 2-way ANOVA revealed no significant associations (Multimedia Appendix 1) [45].

Parents’ average Chew and NVS scores were 2.5 (SD 2.0) and 4.4 (SD 1.8), respectively, indicating adequate general health literacy in the total sample. Parental self-efficacy regarding diabetes management was generally high, with an average PSESDM score of 32.9 (SD 5.4). The average HFS score was 33.7 (SD 12.3).

Subgroup comparisons by sociodemographic characteristics revealed that parents’ health literacy (eHEALS, Chew, and NVS) and self-efficacy (PSESDM) differed significantly by their educational level but not their fear of hypoglycemia (HFS) (Table 1).

### Children’s T1DM Disease Characteristics and HRQoL

In total, 150 children (girls: 69/150, 46.0%) were included, with a mean disease duration of 5.3 (SD 2.8) years. Their mean age, height, and weight were 11.7 (SD 1.9) years, 150.6 (SD 16.4) cm, and 45.9 (SD 16.3) kg, respectively. Altogether, 106 (70.7%) children used a glucose sensor. The most frequent insulin treatment modality was pen plus sensor, followed by pump plus sensor, pen, and pump (Table 2).

**Table 2:** Characteristics of children with type 1 diabetes mellitus and diabetologists’ perceptions in the total sample and by treatment modality.

Variable	Total sample (N=150)	Pen (n=38)	Pen + sensor (n=55)	Pump (n=6)	Pump + sensor (n=51)	*P* value^a^
Age (years), mean (SD)	11.7 (1.9)	11.7 (2.0)	11.9 (1.9)	11.3 (1.6)	11.6 (1.8)	.84
Disease duration (years), mean (SD)	5.3 (2.8)	5.4 (3.1)	4.2 (2.3)	7.2 (2.8)	6.2 (2.8)	<.001
Duration of care at the center (years), mean (SD)	4.8 (2.6)	5.2 (2.7)	3.7 (2.3)	7.0 (2.7)	5.5 (2.3)	<.001
Current HbA_1c_ (%)^b^, mean (SD)	7.6 (1.4)	8.6 (2.0)	7.4 (1.1)	7.2 (0.5)	7.2 (0.8)	<.001
6-month average HbA_1c_ (%)^b^, mean (SD)	7.6 (1.3)	8.5 (1.9)	7.3 (1.0)	7.2 (0.5)	7.2 (0.7)	<.001
PedsQL^c^ (range: 0-100), mean (SD)	81.4 (13.2)	78.3 (17.8)	81.5 (12.0)	86.4 (14.0)	82.9 (9.7)	.30
PedsQL physical subscore (range: 0-100), mean (SD)	85.7 (13.0)	82.9 (17.6)	87.0 (11.7)	86.5 (11.8)	86.3 (10.2)	.49
PedsQL psychosocial subscore (range: 0-100), mean (SD)	79.1 (14.7)	75.8 (19.6)	78.6 (13.6)	86.4 (16.1)	81.2 (10.8)	.22
PedsQL Diab^d^ (range: 0-100), mean (SD)	74.4 (12.8)	72.0 (15.7)	73.8 (11.6)	84.4 (10.1)	75.7 (11.5)	.13
EQ-5D-Y-3L index (range: −0.485 to 1.000), mean (SD)	0.940 (0.097)	0.915 (0.119)	0.939 (0.097)	0.964 (0.058)	0.957 (0.077)	.21
Parents’ cooperation (VAS^e,f^), mean (SD)	7.1 (2.4)	4.7 (2.0)	7.7 (2.1)	6.5 (1.6)	8.3 (1.7)	<.001
Parents’ diabetes knowledge (VAS^f^), mean (SD)	6.9 (2.3)	4.6 (1.7)	7.5 (1.9)	5.7 (1.5)	8.1 (1.6)	<.001
Parents’ device use knowledge (VAS^f^), mean (SD)	6.2 (2.7)	2.7 (1.6)	7.1 (1.9)	5.5 (1.0)	7.9 (1.7)	<.001
T1DM^g^ being difficult to treat (VAS^f^), mean (SD)	6.6 (2.4)	4.3 (2.0)	7.2 (2.2)	5.8 (1.3)	7.7 (1.8)	<.001

^a^Differences between treatment modalities were compared with ANOVA.

^b^A higher HbA_1c_ level indicates worse glycemic control.

^c^PedsQL: Pediatric Quality of Life Inventory.

^d^PedsQL Diab: Pediatric Quality of Life Inventory Diabetes Module.

^e^VAS: visual analog scale.

^f^Based on diabetologists’ assessments. Lower scores indicate worse cooperation and knowledge, and more difficulties in treatment.

^g^T1DM: type 1 diabetes mellitus.

The average EQ-5D-Y-3L index and PedsQL score in the sample were 0.940 (SD 0.097) and 81.4 (SD 13.2), respectively, indicating that the general HRQoL of children living with T1DM was high. The PedsQL Diab score was moderately low (mean 74.4, SD 12.8).

Any type of comorbidity was observed in 43 children (29 had T1DM-related thyroid disease; 12 had coeliac disease; and 1 each had growth hormone deficiency, juvenile idiopathic arthritis, congenital adrenal hyperplasia, and epilepsy). An acute event requiring a physician or a device malfunction in the past 3 months was reported in 4 children (3 had a severe hyperglycemic episode or ketoacidosis and 2 had device malfunction). T1DM-related chronic kidney complication was noted in 1 child.

Children’s characteristics and differences by treatment modality are presented in Table 2. Both disease duration and time of care in the pediatric diabetology center were the longest among patients using an insulin pump (without sensor), while the highest average HbA_1c_ levels were observed in the subgroup using a pen (without sensor). HRQoL results (PedsQL, PedsQL Diab, and EQ-5D-Y-3L) did not differ significantly across treatment modality subgroups. No meaningful difference in the occurrence of comorbidities was found across treatment types.

### Diabetologists’ Perceptions of Parents and Disease Management

In the total sample, the average scores for parents’ cooperation, diabetes knowledge, device use knowledge, and difficulty in managing the child’s disease were 7.1 (SD 2.4), 6.9 (SD 2.3), 6.2 (SD 2.7), and 6.6 (SD 2.4), respectively. The relationship of these characteristics with parental age showed a nonsignificant concave pattern (Multimedia Appendix 2). Parents’ cooperation with their child’s diabetes management and disease-related knowledge (both of diabetes and device use) significantly differed by treatment modality (being the highest in the pump + sensor subgroup, followed by the pen + sensor subgroup). Treating the child’s T1DM was found to be the least difficult in the pump + sensor subgroup and the most difficult in the pen (without a sensor) subgroup (Table 2).

### Analysis by Treatment Modality and T1DM Complications

Parents’ health literacy and attitudes toward their child’s diabetes by major subgroups are presented in Table 3. The eHEALS score differed significantly by treatment modality, being the highest in the pump + sensor subgroup, followed by the pen + sensor, pen (without sensor), and pump (without sensor) subgroups. However, no differences were detected in terms of the occurrence of serious acute events, device malfunction, or prevalent comorbidities.

**Table 3:** Parents’ health literacy and attitudes toward their child’s diabetes by major subgroups.

Variable		Value (N=150), n	eHEALS^a^			Chew^b^				NVS^c^					PSESDM^d^					HFS^e,f^				
			Score, mean (SD)	*P* value^g^	Score, mean (SD)		*P* value^g^		Score, mean (SD)			*P* value^g^	Score, mean (SD)			*P* value^g^		Score, mean (SD)			*P* value^g^			
**Treatment modality**				*.*005			*.*09					*<.*001				*.*002					*.*32			
	Pen	38	29.5 (5.0)		3.2 (2.5)				3.4 (2.0)					30.3 (5.7)					33.5 (12.7)					
	Pen + sensor	55	31.7 (5.0)		2.4 (1.8)				4.6 (1.6)					32.9 (5.2)					35.0 (11.8)					
	Pump	6	27.0 (5.3)		2.7 (1.9)				3.8 (2.6)					33.0 (4.9)					39.8 (7.3)					
	Pump + sensor	51	32.4 (4.0)		2.2 (1.7)				5.1 (1.3)					34.7 (4.7)					31.6 (13.0)					
**Any acute T1DM^h^-related event requiring medical intervention or a device malfunction in the last 3 months**				*.*34			*.*35					*.*04				*.*31					*.*049			
	Yes^i^	4	29.8 (2.6)		1.5 (1.9)				1.5 (1.7)					28.5 (7.3)					27.8 (4.3)					
	No	146	31.2 (4.9)		2.6 (2.0)				4.5 (1.7)					33.0 (5.3)					33.8 (12.5)					
**Any comorbidity**				*.*76			*.*87					*.*64				*.*93					*.*47			
	Yes	43	31.0 (5.0)		2.6 (1.8)				4.5 (1.6)					32.8 (5.5)					32.6 (10.4)					
	No	104	31.3 (4.9)		2.5 (2.1)				4.4 (1.8)					32.8 (5.4)					34.1 (13.2)					
Total sample			31.2 (4.9)		2.5 (2.0)				4.4 (1.8)					32.9 (5.4)					33.7 (12.3)					

^a^eHEALS: eHealth Literacy Scale.

^b^Higher scores indicate lower literacy levels.

^c^NVS: Newest Vital Sign.

^d^PSESDM: Parental Self-Efficacy Scale for Diabetes Management.

^e^HFS: Hypoglycemia Fear Survey.

^f^Higher scores indicate greater fear of hypoglycemia.

^g^Differences between groups were compared using Welch and ANOVA tests.

^h^T1DM: type 1 diabetes mellitus.

^i^The events reported by the diabetologists were hyperglycemia or ketoacidosis requiring medical intervention and device malfunction.

Parents’ NVS and PSESDM scores differed significantly by treatment modality, but there were no differences in the Chew and HFS scores. Parents whose children experienced any T1DM-related serious acute event or device malfunction in the last 3 months had lower general health literacy (NVS) and lower fear of hypoglycemia (HFS). The Chew score showed no significant difference by subgroups.

### Correlations Between eHEALS and Other Measures

**Figure 2:**
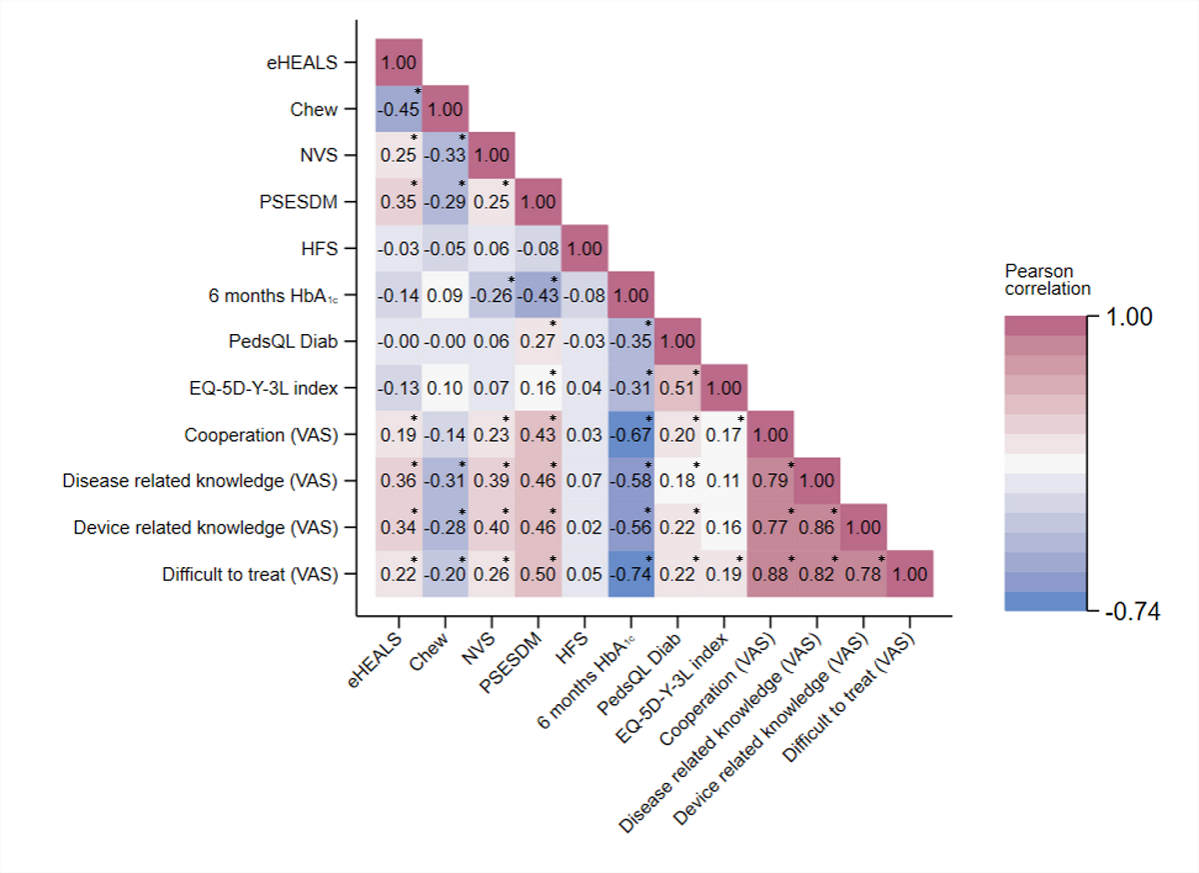
Correlation of parents’ electronic health literacy with child-related outcome measures and diabetologists’ assessment scores (Pearson correlation). eHEALS: eHealth Literacy Scale; HFS: Hypoglycemia Fear Survey; NVS: Newest Vital Sign; PedsQL Diab: Pediatric Quality of Life Inventory Diabetes Module; PSESDM: Parental Self-Efficacy Scale for Diabetes Management; VAS: visual analog scale. *P<.05.

The correlation of the parental eHEALS score was moderate with the Chew score (r=−0.45; *P*<.001) and weak with the NVS score (r=0.25; *P*=*.*002). Moreover, a moderate positive correlation was seen with the PSESDM score (r=0.35; *P*<.001) but not with the HFS score (r=−0.03; *P*=.70). Regarding children’s T1DM outcomes, the parental eHEALS score did not correlate significantly with children’s 6-month HbA_1c_ level (r=−0.143; *P*=.08) and HRQoL outcomes (PedsQL Diab: r=−0.0002; *P*>.99; EQ-5D-Y-3L: r=−0.13; *P*=.12). Significant but low or moderate correlations were observed between the eHEALS score and how diabetologists perceived parents’ cooperation (r=0.19; *P*=.02), diabetes knowledge (r=0.36; *P*<.001), device use knowledge (r=0.34; *P*<.001), and level of difficulty in managing the disease (r=0.22; *P*=.008). The results are shown in Figure 2.

### Regression Results

The determinants of glucose control in different regression models are presented in Table 4. Neither parental electronic and general health literacy (eHEALS, Chew, and NVS) nor fear of hypoglycemia (HFS) showed a significant association with the child’s 6-month HbA_1c_ level. The final model (Model 9) that included all variables explained 47.0% of the total variance in HbA_1c_, which was significantly associated with the child’s sex (girls having a higher HbA_1c_ compared to boys), treatment modality (pen + sensor and pump + sensor users having a lower HbA_1c_ compared to pen users), and parental self-efficacy in managing their child’s diabetes (PSESDM; a higher parental self-efficacy was associated with a lower child HbA_1c_ level).

**Table 4:** Determinants of glucose control (6-month average HbA_1c_) in different regression models (N=150).

Variable		Model								
		M1	M2	M3	M4	M5	M6	M7	M8	M9
Age (child)		0.051	0.121	0.096	0.091	0.099	0.096	0.091	0.096	0.082
**Sex (child) (reference: boy)**										
	Girl	0.277	0.512^a^	0.469^a^	0.495^a^	0.459^a^	0.470^a^	0.550^b^	0.467^a^	0.578^b^
T1DM^c^ duration		−0.037^d^	0.105	0.100	0.093	0.112	0.103	0.104	0.100	0.102
Duration of care at the center		0.199^a^	0.084	0.106	0.120	0.097	0.105	0.089	0.107	0.100
Age (parent)		—^e^	−0.007^d^	−0.016^d^	−0.014^d^	−0.012^d^	−0.016^d^	−0.026^d^	−0.016^d^	−0.023^d^
**Sex (parent) (reference: male)**										
	Female	—	−0.051^d^	−0.110^d^	−0.085^d^	−0.117^d^	−0.110^d^	−0.149^d^	−0.108^d^	−0.109^d^
**Education (reference: primary)**										
	Secondary	—	−0.851^a,d^	−0.364^d^	−0.357^d^	−0.318^d^	−0.370^d^	−0.379^d^	−0.362^d^	−0.321^d^
	Tertiary	—	−1.369^b,d^	−0.731^d^	−0.762^d^	−0.768^d^	−0.751^d^	−0.706^d^	−0.726^d^	−0.762^d^
**Residence (reference: capital)**										
	Town	—	−0.178^d^	0.001	−0.026^d^	0.005	−0.004^d^	0.037	−0.001^d^	−0.011^d^
	Village	—	−0.285^d^	−0.123^d^	−0.178^d^	−0.105^d^	−0.119^d^	−0.131^d^	−0.123^d^	−0.181^d^
Income		—	−0.001^d^	0.000	0.000	0.000	0.000	0.000	0.000	0.000
**Living in the same household (reference: full-time)**										
	Part-time	—	−0.207^d^	−0.158^d^	−0.198^d^	−0.082^d^	−0.170^d^	−0.282^d^	−0.161^d^	−0.322^d^
**Treatment modality (reference: pen)**										
	Pen + sensor	—	—	−0.745^a,d^	−0.754^a,d^	−0.754^a,d^	−0.751^a,d^	−0.643^a,d^	−0.746^a,d^	−0.660^a,d^
	Pump	—	—	−1.269^a,d^	−1.149^d^	−1.214^a,d^	−1.271^a,d^	−1.240^a,d^	−1.270^a,d^	−1.018^d^
	Pump + sensor	—	—	−1.081^b,d^	−1.081^b,d^	−1.097^b,d^	−1.091^b,d^	−0.919^b,d^	−1.083^b,d^	−0.927^b,d^
eHEALS^f^		—	—	—	0.024	—	—	—	—	0.034
Chew		—	—	—	—	−0.063^d^	—	—	—	−0.061^d^
NVS^g^		—	—	—	—	—	0.012	—	—	0.015
PSESDM^h^		—	—	—	—	—	—	−0.068^b,d^	—	−0.082^b,d^
HFS^i^		—	—	—	—	—	—	—	0.000	−0.004^d^
Constant		6.074^j^	6.744^j^	7.264^j^	6.511^j^	7.259^j^	7.232^j^	9.742^j^	7.281^j^	9.317^j^
R^2^		0.126	0.328	0.396	0.402	0.403	0.396	0.444	0.396	0.470

^a^*P*<.05.

^b^*P*<.01.

^c^T1DM: type 1 diabetes mellitus.

^d^Negative coefficients represent a decrease in the HbA_1c_ level for a 1 unit increase in a given variable, which consequently represents an improvement in glucose control.

^e^Variable was not part of the model.

^f^eHEALS: eHealth Literacy Scale.

^g^NVS: Newest Vital Sign.

^h^PSESDM: Parental Self-Efficacy Scale for Diabetes Management.

^i^HFS: Hypoglycemia Fear Survey.

^j^*P*<.001.

## Discussion

In this cross-sectional clinical study, we investigated the electronic health literacy of parents caring for children with T1DM, using the eHEALS self-reported measurement tool, and results were analyzed alongside their general health literacy. Associations of eHEALS with disease management and disease outcomes were also investigated. On the eHEALS questionnaire, parents reported substantial problems with finding, understanding, and using electronic health information. Regarding disease management, eHEALS scores differed significantly according to the children’s treatment modality, being the highest in the pump + sensor subgroup, and there was a significant association of eHEALS scores with parents’ self-efficacy in managing diabetes (PSESDM) and the diabetologists’ perceptions of parents as T1DM caregivers. Regarding disease outcomes, we found no significant associations with parental eHEALS scores. Regression analysis revealed that the 6-month average HbA_1c_ level was associated with the child’s sex, treatment modality, and PSESDM score, but not with the electronic and general health literacy scores. To our knowledge, this is the first study to investigate parental electronic health literacy (eHEALS) in pediatric T1DM.

Comparisons with the international literature are hampered by the lack of electronic health literacy studies in this patient group and the variability of the general health literacy measurement tools used. In our study, more educated parents had significantly higher electronic (eHEALS) and general (Chew and NVS) health literacy, and these 2 differed significantly by income level as well. In contrast, previous studies involving young children [31] and adolescents [40] have reported no significant differences in parents’ general health literacy (assessed by the S-TOFHLA) by sociodemographic subgroups. Moreover, Al-Abdulrazzaq et al [41] found no association between parents’ NVS score and their educational level in a validation study of the Arabic version of the NVS. Parental self-efficacy in the child’s diabetes management (PSESDM) showed an increasing trend by educational level and income in our study, but parental fear of hypoglycemia (HFS) did not differ by sociodemographic subgroups. Marchante et al [51] reported that PSESDM was associated with the child’s sex. In the study by Amiri et al [63], neither parental self-efficacy (assessed by the Self-Efficacy for Diabetes Scale-Parent questionnaire) nor the HFS score differed significantly by demographic characteristics. These controversial results regarding the role of sociodemographics in parental health literacy and caregiver attitude need further investigation in large epidemiological studies. We consider it important to highlight the female dominance of parents (80%) in our sample. We acknowledge that we could have obtained different results in other care settings and that the child’s T1DM might affect the quality of life and employment perspectives of other family members. These points definitely deserve further exploration. Nevertheless, our results suggest that mothers play a key role in the T1DM care of their children. It is therefore worth paying particular attention to their electronic health literacy, capability, and willingness to operate modern devices, considering their preferences, and measuring how they can benefit from new digital technologies.

Our study revealed significantly higher parental electronic health literacy (eHEALS) in children using a digital sensor to measure their blood glucose level. The results suggest that parental electronic health literacy might affect the choice of insulin administration and glucose measurement mode. This choice may depend on various factors, including the judgement of the treating diabetologist, reimbursement rules, availability of devices, and access to devices, as well as on the acceptability of different treatment modalities (ie, parental consent and the child’s preferences). Further studies involving pediatric diabetes care providers from different levels and diverse patient groups are encouraged to explore in depth the decision-making process. Parents’ average NVS score also differed significantly by treatment groups, suggesting that general health literacy (focusing on skills for both words and numbers) might play a significant role in treatment decisions. This is in contrast with findings by Pulgaron et al [31], as parents’ reading and numeracy abilities were not associated with treatment modalities. However, it is important to note that much younger (aged 3-9 years; mean age 6.8 years) T1DM children were involved in their study.

HbA_1c_ is an important indicator of T1DM management. We found no significant correlation with parental eHEALS and Chew scores, but there were weak and moderate relationships with parental general health literacy (NVS) and self-efficacy (PSESDM), respectively. In the study by Pulgaron et al [31], parents’ numeracy skills were negatively correlated with the child’s HbA_1c_ level (r=−0.52), which strengthens our results with the NVS measure that also has a strong numerical focus. However, 2 other studies found no association between parental health literacy (S-TOFHLA and NVS) and the child’s HbA_1c_ level [40,41]. Ross et al [43] reported that glycemic disease control was worse for those children whose caregivers had lower literacy skills as measured by the National Adult Reading Test (NART).

An important observation of our study was that parental electronic and general health literacy scores and parental fear of hypoglycemia were not associated with the child’s HbA_1c_ level in the regression analyses, and contributed minimally to the total variance explained (R^2^), indicating that these factors have a negligible effect on the child’s glucose control. We also found that apart from the child’s sex and parents’ PSESDM score, only pen + sensor and pump + sensor treatment modalities remained significantly associated with the HbA_1c_ level in the final regression model, when all relevant variables were added together. Thus, a digital glucose sensor can have a meaningful positive effect on the child’s glucose control. Our observations are consistent with previously published results. In most previous studies, higher HbA_1c_ levels were found among female young children than among male children both at diagnosis and during treatment [64]. Pulgaron et al [31] reported that parents’ numeracy skills could significantly predict HbA_1c_, but the significance was lost when parents’ education was added as a covariate in the regression analysis. In their final model, only parental self-efficacy regarding diabetes management (Perceived Diabetes Self-Management Scale) remained a significant predictor of HbA_1c_. Furthermore, Al-Abdulrazzaq et al [41] found that adequate health literacy was inversely associated with optimal HbA_1c_ levels, but adjusting for the treatment regimen cancelled its significant effect. We think, however, that the child’s HbA_1c_ level is multifactorial, and not all relevant factors were taken into account in our research. For instance, at this university-based center, patients are closely monitored and have opportunities for consultation with highly qualified pediatric diabetologists and a multidisciplinary team. This tight control may partly balance the differences in parental disease management capabilities in terms of HbA_1c_ outcome. It is necessary to note that we ran the regression for HbA_1c_, but it is not the only significant outcome of the disease. The benefits of new digital technologies, if used by properly trained users with great digital skills, can also be seen in areas not covered in our study. For instance, the possibility of remote control of the child’s status might put the parents into a much better position in terms of feeling more safe and flexible in managing other family members and their own life. It would be worth also investigating how the electronic health literacy of main caregivers (mothers in our study) impacts long-term management decisions and disease outcomes in the patient’s later adolescence and adulthood.

Some limitations of our study have to be mentioned. First, this was a single-center cross-sectional study in a university clinic, which limits the generalizability of our results. It would be interesting to investigate whether the role of eHEALS for HbA_1c_ levels is similarly negligible in jurisdictions where parents have less access to resources and high-quality, personalized, family-centered diabetology care [65], and are more reliant on information from the internet. It would be worthwhile to also assess in a follow-up study how parents’ digital health literacy changes with digital device use and participation in diabetes care. Second, the tools used to measure parental health literacy were not specific to diabetes. Therefore, we may not have been able to capture all relevant aspects of parental knowledge. Third, only 4 children had serious acute events requiring medical intervention or device malfunction in the past 3 months. Hence, the importance of parental electronic health literacy in acute events needs further research. Fourth, only 6 children used an insulin pump without a sensor, which limits the generalizability of the results of this subgroup. Our study showed that disease duration and follow-up at this university-based clinic were the longest for this subgroup. Discussions with treating diabetologists confirmed that these patients have usually been using pump treatment for a long time and often struggle with switching from conventional blood glucose measurement to sensor measurement.

This first exploratory study provides insights into the electronic health literacy of parents caring for their child with T1DM. Parents’ educational level was identified as an important sociodemographic factor affecting parental electronic health literacy and attitudes toward their child’s diabetes. Meaningful differences in parental eHEALS and NVS scores were found by treatment modality, suggesting that parental electronic and general health literacy may be important factors in treatment decisions. In addition to being a male child, higher parental self-efficacy in diabetes management and sensor use were positively associated with better child glucose control. However, a lack of a direct association between this core outcome and parental electronic and general health literacy raises the issue of further influencing factors not considered in this study, as well as the need for diabetes-specific electronic health literacy measurement tools. Further prospective multicenter studies involving heterogeneous settings and care providers are recommended to strengthen and refine our observations.

## Appendix

Multimedia Appendix 1 Comparison of eHealth Literacy Scale scores observed in the current study and the Hungarian population sample.

Multimedia Appendix 2 Diabetologists’ perceptions of parental cooperation, diabetes knowledge, device use knowledge, and difficulty of disease management by the parents’ age group.

## Abbreviations

eHEALS: eHealth Literacy Scale
HFS: Hypoglycemia Fear Survey
HRQoL: health-related quality of life
NVS: Newest Vital Sign
PedsQL: Pediatric Quality of Life Inventory
PedsQL Diab: Pediatric Quality of Life Inventory Diabetes Module
PSESDM: Parental Self-Efficacy Scale for Diabetes Management
S-TOFHLA: Short Test of Functional Health Literacy in Adults
T1DM: type 1 diabetes mellitus
VAS: visual analog scale

## Data Availability

The data supporting the findings of this study are available from the corresponding author upon request.
