# Supplementary material for: The Impact of Parental Electronic Health Literacy on Disease Management and Outcomes in Pediatric Type 1 Diabetes Mellitus: Cross-Sectional Clinical Study
Source: JMIR Pediatr Parent. 2024 Mar 20;7:e54807. doi: 10.2196/54807 (PMC10993131; doi:10.2196/54807)
Supplement: Multimedia Appendix 1 [file pediatrics_v7i1e54807_app1.docx]

**Multimedia Appendix 1.** Comparison of eHealth Literacy Scale scores observed in the current study and the Hungarian population sample.

|  | **Current study** | | **Hungarian population sample* [45]** | | **p**** |
| --- | --- | --- | --- | --- | --- |
|  | **N** | **eHEALS score**  ***mean (SD)*** | **N** | **eHEALS score**  ***mean (SD)*** |  |
| **Total sample** | 150 | 31.2 (4.9) | 1000 | 29.2 (5.2) | <0.001 |
| **Sex** | | | | |  |
| *Men* | 30 | 31.8 (4.3) | 450 | 28.8 (5.6) | 0.935 |
| *Women* | 120 | 31.0 (5.0) | 550 | 29.5 (4.8) |  |
| **Age group** | | | | |  |
| *18-24* | 1 | 29.0 (0.0) | 118 | 29.5 (5.2) | 0.580 |
| *25-34* | 13 | 29.5 (6.1) | 198 | 30.0 (5.2) |  |
| *35-44* | 72 | 31.8 (4.5) | 191 | 29.7 (5.3) |  |
| *45-54* | 62 | 30.9 (5.0) | 125 | 29.0 (5.1) |  |
| *55-64* | 2 | 29.0 (1.4) | 147 | 28.4 (4.7) |  |
| *65+* | - | - | 221 | 28.4 (5.3) |  |
| **Education***** | | | | |  |
| *Primary* | 15 | 29.5 (5.6) | 341 | 28.8 (5.3) | 0.115 |
| *Secondary* | 70 | 30.5 (4.7) | 363 | 29.3 (4.8) |  |
| *Terciary* | 64 | 32.3 (4.7) | 296 | 29.3 (5.5) |  |

*Person level data for the comparisons were provided by the authors)

**Differences were examined by Welch and two-way ANOVA tests.

***There was 1 missing value for education in the current study.
