# Supplementary material for: The Impact of Parental Electronic Health Literacy on Disease Management and Outcomes in Pediatric Type 1 Diabetes Mellitus: Cross-Sectional Clinical Study
Source: JMIR Pediatr Parent. 2024 Mar 20;7:e54807. doi: 10.2196/54807 (PMC10993131; doi:10.2196/54807)
Supplement: Multimedia Appendix 2 [file pediatrics_v7i1e54807_app2.docx]

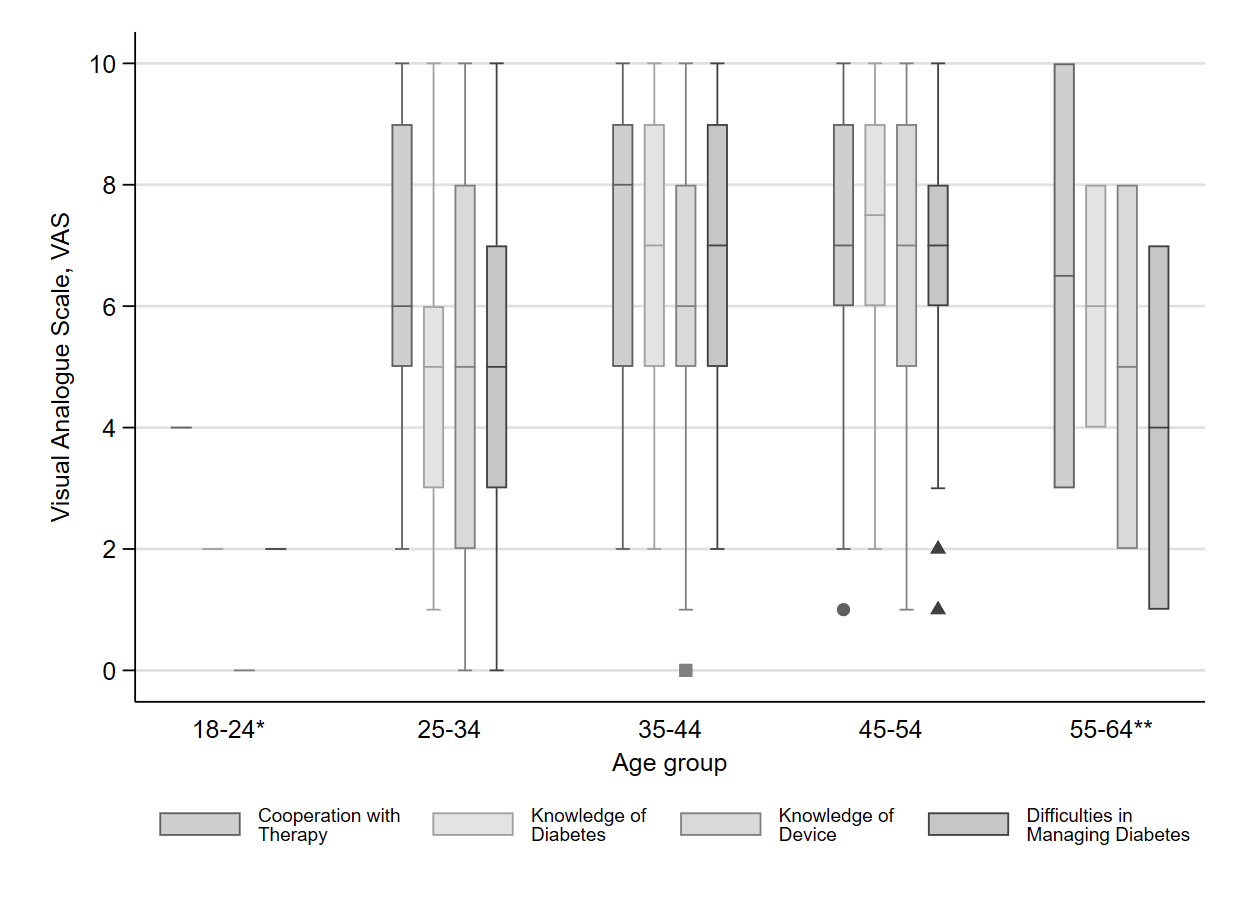


**Multimedia Appendix 2.** Diabetologists’ perceptions of parental cooperation, diabetes knowledge, device use knowledge, and difficulty of disease management by the parents’ age group. Lines in the middle of boxes indicate medians, upper and lower hinges indicate 75th and 25th percentiles, and whiskers with adjacent lines indicate adjacent values; *There was 1 parent in the age group 18-24; **There were 2 parents in the age group 55-64.
